# Effectiveness of cognitive analytic therapy for bipolar affective disorder: A co‐produced single subject cumulative treatment design with extended follow‐up (A^1^/B/A^2^/C‐FU)

**DOI:** 10.1111/papt.12390

**Published:** 2022-03-10

**Authors:** Stephen Kellett, Lisa Alhadeff, Chris Gaskell, Melanie Simmonds‐Buckley

**Affiliations:** ^1^ University of Sheffield and Sheffield Health and Social Care NHS Foundation Trust Sheffield UK; ^2^ Patient Participant Sheffield Health and Social Care NHS Foundation Trust Sheffield UK; ^3^ University of Sheffield Sheffield UK

**Keywords:** bi‐polar, CAT, co‐production, SCED

## Abstract

**Objectives:**

Evidence for the treatment of bipolar affective disorder with cognitive analytic therapy (CAT) is limited, and so this study sought to intensively evaluate outcomes in a co‐produced single‐case experimental design (SCED).

**Design:**

An A^1^/B/A^2^/C with extended follow‐up SCED with a female patient meeting diagnostic criteria for bipolar disorder.

**Methods:**

Following the 6‐week baseline period ‘A^1^’, treatment occurred in two phases (18 ‘B’ and 6 sessions ‘C’) sandwiching a 12‐week treatment withdrawal phase (‘A^2^’) and a 24‐week structured follow‐up phase. Five idiographic daily measures were collected daily to create a 622‐day timeline. The PHQ‐9 and the Mania Rating Scale were completed after each treatment session. The participant held two roles: as the patient and provider of the idiographic/nomothetic outcomes and also as part of the research team through providing a commentary on the outcomes identified.

**Results:**

CAT was a partially effective intervention. There were improvements to idiographic measures of self‐criticism, self‐acceptance, body dissatisfaction and worry. Nomothetic outcomes showed little change. CAT did not insulate from the occurrence of a hypermanic relapse during the follow‐up phase. The change commentary mirrored the idiographic outcomes in noting that the ‘exits’ were harder to implement during the manic relapse.

**Conclusions:**

This co‐produced SCED suggests a partially effective CAT intervention, but with exits much harder to sustain during manic relapse. Methodologically, it is possible to improve SCED methodology through widening the participant role further beyond that of data collection.

**Practitioner points**

- Taking a relational approach to treating bipolarity is possible, as the differing mood states create and maintain differing relational patterns.
- Therapists need to complete structured follow‐up with bipolar patients to capture any emerging relapse.
- Intensively evaluating therapies and co‐producing outcome research with patient participants is clearly possible when underpinned by negotiation, mutuality and compromise, but might not suit all patients.

## INTRODUCTION

Bipolar affective disorder is defined by the presence of recurrent and episodic periods of mania or hypomania and periods of profound depression, which predict clinically significant shifts in energy/activity levels to a level that disrupts functioning (American Psychiatric Association, 2013). Bipolar affective disorder is equally distributed across the genders and has a lifetime prevalence of 1.3%–1.6% (NIMH, 2012). Functioning is disrupted as patients spend as much as 47% of their adult lives in manic or depressed states (Judd et al., 2002). After a manic episode, 50% of patients have not recovered within a year and only 25% then go onto to achieve full recovery of functioning (Keck et al., 1998). The relapse rate for bipolar affective disorder is therefore high (71%; Belete et al., 2020). Lithium (and other mood stabilizing medications) offers pharmacological support (Young & Hammond, 2007), but adherence to these treatment regimens can be piecemeal (i.e. 20%–60% of cases; Adams & Scott, 2000). Responsivity to treatment is negatively affected when there is comorbidity with other psychiatric disorders, particularly that of personality disorder (Latalova et al., 2013). In terms of interplay of personality and bipolar disorder, then earlier childhood trauma generates more severe manic and depressed states and increases risk of suicide and substance misuse (Aas et al., 2016).

The evidence base for adjunctive psychotherapy for BD consists of six differing treatments (see Salcedo et al., 2016 for a review): psychoeducation, cognitive behavioural therapy, interpersonal and social rhythm therapy, dialectical behaviour therapy, mindfulness based cognitive therapy and family therapy. Meta‐analyses have shown that adjunctive psychotherapy can significantly reduce relapse rates (Scott et al., 2007) and enhance symptomatic and functional outcomes over 2‐year periods (Miklowitz, 2008). Family therapy, interpersonal therapy and systematic care appear most effective in preventing relapse and cognitive–behavioural therapy and group psychoeducation appear most effective when delivered during a period of recovery (Miklowitz, 2008). All the reviews have called for the development of other psychological treatments in order to improve patient choice for bipolar patients, but have also underlined the need for thorough empirical evaluation.

Cognitive analytic therapy (CAT) is a popular integrative therapy distinct in its explicit relational focus and methods (Ryle & Kerr, 2003), with a well‐defined competency model (Parry et al., 2021), associated competency measure (Bennett & Parry, 2004) and increasingly convincing evidence base (Hallam et al., 2021). CAT is typically used to treat complex and enduring mental health problems in Secondary Care services, delivered via 24 weekly one‐to‐one sessions plus 6 months of structured follow‐up (Ryle et al., 2014). CAT for bipolar affective disorder is underpinned by use of the multiple self‐states model (MSSM; Ryle & Marlowe, 1995), as this formulates both the manic and depressed elements of the presentation and their associated opposing relational dynamics (Fountouakis, 2008; Shannon & Swarbrick, 2010). The evidence of CAT for bipolar affective disorder is limited to two previous group studies. Kerr (2001) reported on a case series (*N* = 4) in which two of the patients had a good qualitative outcome. Evans et al. (2017) conducted a pilot randomized controlled trial in which patients either received CAT (*N* = 9) or treatment as usual (*N* = 9). No adverse events occurred, 8/9 completed treatment, 5/8 attended all sessions and 2/8 were categorized as recovered in the CAT‐arm. No differences on nomothetic outcomes occurred between the arms.

An unfortunate consequence of group studies such as the Evans et al. (2017) trial is that mean outcome scores on nomothetic measures obscures both the responsivity (or not) of individuals and also whether the idiosyncratic problems brought by patients to therapy actually change (Heneghan et al., 2017). Single‐case experimental designs (SCED) address this issue by focussing on individual patients, their idiosyncratic problems and analysing responsivity to treatment of associated idiographic measures (Barlow et al., 2009). The defining features of SCED are as follows: (1) repeated and very intensive sampling of ideographic measures that always start with a baseline and against which intervention phases are compared, (2) manipulation of one or more independent variables whilst controlling for sources of bias and (3) demonstration of stability within and across levels of imposed independent variables (Kratochwill et al., 2013). Lillie et al. (2011) described SCED as a key strategy for individualizing medicine. There have been no previous SCED studies of CAT for bipolar affective disorder, and so this study sought to conduct a study with high internal validity through conducting a withdrawal experimental design (Barlow et al., 2009). One previous CAT SCED withdrawal design has been conducted with borderline personality disorder (Kellett et al., 2021) that showed a moderately effective intervention. There have been previous calls for more use of withdrawal designs in the CAT SCED evidence base (Kellett & Lees, 2019). It is questionable whether psychotherapeutic treatments that are not purely behavioural, once delivered, can be removed. Although treatment can be halted, some degree of learning shall be assimilated during psychotherapy and therefore can endure beyond termination; this is emphasized within more analytically focused treatments. Based on this rationale, the design of the current study may be more appropriately described as cumulative treatment design (A‐B‐A‐C) as opposed to a true reversible treatment design.

Withdrawal designs (Hersen, 1990) measure a baseline phase in ideographic measures (A^1^ in the current study), a treatment phase (the B^1^ in the current study), the withdrawal of treatment (A^2^ in the current study) and the reintroduction of treatment (the C in the current study). Previous CAT SCED studies have treated the assessment phase as the baseline and this has been challenged as there is contact with the therapist. So, this study sought to innovatively test whether assessment sessions changed idiographic measures through conducting a two‐phase baseline (i.e., containing no therapist contact vs. therapist contact via assessment). Additionally, this study also sought to enable a co‐production of the SCED with the patient participant. Co‐production in the current context was defined as requiring the stakeholders in the study (i.e., the therapist, research team and the patient) to collaboratively work together to achieve the outcome of producing a SCED study. No SCEDs have been co‐produced previously despite the benefits of co‐production being made clear (Lwembe et al., 2017). The hypotheses for the current study were as follows: (1) there would be significant improvements in idiographic measures during active treatment phases compared to baseline and withdrawal, (2) positive changes in the ideographic measures would be sustained over the follow‐up period, and (3) there would be a clinical and reliably significant change on the two nomothetic outcome measures.

## METHOD

### Design

The reporting of this study is based on the single‐case reporting guidelines (SCRIBE; Tate et al., 2016) and the participant provided consent for the study to be conducted and reported (Cooper et al., 2005). Ethical approval was granted (ref: 041077). The co‐production was enabled through the drafting and sharing of the empirical report to enable a shared and balanced account of the therapy and reporting of the outcomes to be created. Three iterations were made to the report as part of the co‐production. The study itself used an A^1^/B/A^2^/C design, but with an additional 6‐month follow‐up phase to capture potential relapse. Five idiographic measures were completed daily throughout all phases of the study. The baseline phase (A^1^) lasted 44 days and consisted of 14 days of no contact with the therapist after briefly agreeing the idiographic measures and then 30 days containing three assessment sessions. The first treatment phase (B) lasted for 168 days containing 18 treatment sessions. The treatment withdrawal phase (A^2^) lasted 160 days, the second treatment phase (C) spanned 84 days and contained 6 sessions and the follow‐up phase was 166 days. The study therefore constituted a time series of *N* = 622 days housing 5 study phases. In terms of missing idiographic data, then no data were missing from (A^1^) and (B), 28/160 days (17.5%) during (A^2^), 26/84 days (30.95%) during (C) and 5/166 (3.01%) days during the follow‐up phase. Two nomothetic outcome measures were also completed at each session during the two treatment phases to index the presence of depressed and manic mood providing *N* = 24 consecutive measurements. The Patient Health Questionnaire‐9 (PHQ‐9; Kroenke & Spitzer, 2002) was used to measure depression, and the Mania Rating Scale (MRS; Young et al., 1978) was used to measure manic mood (see measures section).

### The patient

The participant was a 28‐year‐old female referred from General Practice. The patient participant did not want to have their family history reported in the empirical report. The participant had a diagnosis of bipolar affective disorder that had been verified across many psychiatric assessments. This was due to the sustained presence over time of two clear mood states of depression (self‐critical, very low in mood/motivation, restlessly agitated and self‐harming) and hypermanic (mood elevated, high activity levels, little need for sleep, flight of ideas, pressure of speech, over spending and impulsive risk taking). Due to the self‐harming during the depressed state, the participant had attracted a previous additional diagnosis of borderline personality disorder. This diagnosis did not inform the current intervention, as both participant and therapist had rejected it. The participant had dropped out of their University undergraduate course due to high academic pressure, and that had triggered the first manic episode. Two inpatient admissions under Section II of the Mental Health Act had occurred. At the time of the therapy starting, the participant was enrolled in a PhD (engineering) and was in a settled and long‐term heterosexual relationship. Throughout the psychological intervention, the participant was taking Lithium 1400 mg, Lamotrigine 300 mg and a low dose of quetiapine (50 mg) to aid sleep. In terms of previous psychological interventions, a course of person‐centred counselling had been provided by University health services. This was described as supportive, but not clinically effective. The referral received for the current study explicitly asked for CAT. The other evidenced‐based approaches for bipolar were available in local services, but the GP referrer believed that due to extensive knowledge and contact with the participant, that the preferred intervention was CAT.

### Treatment

Treatment was delivered in the UK in a tertiary outpatient psychotherapy service provided by the National Health Service. The therapist was a male Consultant Clinical Psychologist and CAT psychotherapist and had clinical supervision provided by a UKCP CAT psychotherapist. Following a screening session, the patient was allocated to the 24‐session version of CAT (Ryle & Kerr, 2003). The patient stated mid‐treatment that they needed to go on an industrial placement as part of their PhD training and presumed that the therapy would stop or be suspended. This serendipitously presented the opportunity for the withdrawal aspect of the method and the patient was informed to keep their idiographic measures throughout the placement (i.e., the treatment withdrawal phase), and then return to treatment. All sessions were weekly and lasted for 50 min. All sessions were attended and conducted by the same therapist regardless of study phase.

The first three sessions were focal to assessment tasks (e.g. taking a history) and so did not contain any treatment elements and culminated in a narrative reformulation (i.e. read to the patient at session four). The narrative reformulation made links between the past and present, named possible enactments and stated the target problems and target problem procedures (Ryle & Kellett, 2018). The target problems (TP) and target problem procedures (TPP) were as follows: TP1: mood variability; TPP1: I am either overdoing it in the midst of mania or I am lost in the midst of depression; TP2: relentless striving; TP2: Never feeling good enough inside creates a feeling of anxiety and dread of failing that drives a relentless pursuit of achievements and excellence. I never stop to acknowledge my achievements or progress and so never feel good enough inside and the cycle starts again; TP3: feelings; TP3: I either suppress my feelings and feel nothing or they come tumbling out in a big rush and I am overwhelmed; TP4: People pleasing; TP4: Learning to be focused on the needs of others as child, leaves me prone to being anxious as to what others think of me as an adult and prone to therefore trying to please them. This means that I neglect myself and my focus is always on the other. As the narrative reformulation was read at session 4 (i.e., signalling the end of the baseline and start of active treatment), this was consistent with previous CAT SCED research (e.g. Kellett et al., 2021).

**FIGURE 1:**
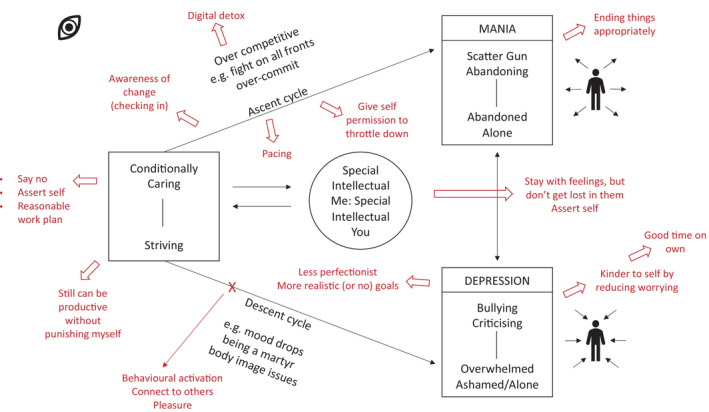
Sequential diagrammatic reformulation (SDR) including exits (in red)

In terms of treatment fidelity, the hallmark components of CAT therapy are a narrative reformulation, a sequential diagrammatic reformulation (SDR) and goodbye letters exchanged at the termination of therapy by patient and therapist (Ryle & Kerr, 2003). In the current case, all these distinctive features of the CAT model were present, and each component was reviewed and apprised at clinical supervision. The manic and depressed states were elicited using the states description procedure (SDP) approach (Ryle, 2007) and a self‐states SDR was co‐produced with the patient (Ryle & Marlowe, 1995). Figure 1 contains the SDR, with the exits that were added in the revision stage of the therapy. CAT is also a therapy that actively works with enactments in the therapeutic relationship (Ryle & Kellett, 2018). The most common enactments were the ‘special me:special you’ enactment and also analysing bullying–victim dynamics in the therapeutic relationship. The goodbye letter by the patient participant noted changes related to reduced striving (i.e., learning how to say no to self and others), being more aware of dynamics of bullying, having processed the trauma of dropping out of University as an undergraduate, climbing differently and for pleasure, more equality in their relationship due to their partner having to care for them less and finally having better boundaries.

### Idiographic and associated analysis strategy

The five idiographic measures were all scored on a 9‐point Likert scale and measured self‐criticism (0 happy with self to 9 extremely self‐critical), self‐acceptance (0 driven to 9 accepting and compassionate), body dissatisfaction (0 hating how my body looks to 9 happy with how my body looks), worrying (0 confident to 9 constantly asking what if) and mood (0 depressed to 9 manic). Comparisons of the trends and differences between the two mini‐phases of the baseline (i.e. no therapist contact vs. therapist contact) were compared and when no differences occurred, collapsed into a single baseline. A time series graph (with lines fitted for individual phase trends, baseline median and lag 7 moving medians to smooth the data and eliminate noise) were created for each idiographic measure. Criterion outlier analysis identified spikes in data points that exceeded two standard deviations from the phase mean (visualized in the time series plots in red), and spike frequency per phase was calculated. Effectiveness of CAT on ideographic outcomes was assessed using a range of non‐overlap statistics: the percentage of data points exceeding the median (PEM; Ma, 2006), the percentage of all non‐overlapping data (PAND; Parker et al., 2007) and non‐overlap of all pairs (NAP; Parker & Vannest, 2009). Non‐overlap outcomes were interpreted as <70% (questionable/ineffective treatment), 70–90% (moderately effective treatment) and >90% (highly effective treatment; Scruggs & Mastropieri, 1998). τU statistics were also used to assess difference between baseline and prospective phases. τU refers to a family of statistics based on Kendall's non‐parametric, rank order coefficient (Brossart et al., 2018). Tau variants were used to assess for: (1) baseline trend (τ); (2) the difference between phases (τU^AvsB^); and (3) the difference between phases when needing to account for significant trend (τU^[AvsB]‐Atrend^). It is recommended that if the baseline trend is not significant, then τU^AvsB^ should be used over τU^(AvsB)−Atrend^ (Brossart et al., 2018). Non‐overlap statistics, τU, outlier analysis and time series plots were all produced using the *Single Case Analysis* package (SCAN; Wilbert & Lueke, 2021) in R (R Core Team, 2021).

### Nomothetic outcome measures and associated analysis strategy

The sessional nomothetic outcomes were graphed and analysed using the reliable change index (RCI; Jacobson & Truax, 1991) and assessed for reliable and clinically significant change (RCSC, Jacobson & Truax, 1991).

#### Patient Health Questionnaire‐9

This 9‐item (0–3) scale (Kroenke & Spitzer, 2002) is based on the nine DSM‐V criteria listed under criterion A for major depressive disorder. The PHQ‐9 is scored as minimal (1–9), mild depressive symptoms (10–14), 15–19 moderate depressive symptoms (15–19) and severe depressive symptoms (20–27) and scores need to reduce by 5 points in order for reliable change to be recorded. The PHQ‐9 is valid and reliable measure of depressed mood and is often used in case identification (Louzon et al., 2016).

#### Altman Mania Scale

This is 5‐item (0–4) scale (Altman et al., 1997) measures presence of positive mood, over confidence, disturbed sleep, speech pattern/amount and also motor activity in the past week. A sum score of more than 6 indicates the presence of a manic or hyper manic mood state (based on a sensitivity rating of 85.5% and a specificity rating of 87.3%). The ATM is reliable and single factor scale measure of mania (Kim & Kwon, 2017).

## RESULTS

Results are presented in four sections: baseline stability, idiographic outcomes across the phases, nomothetic outcomes and then the patient account of change.

### Baseline stability

Table 1 contains the baseline data on the five ideographic measures split between no therapist and therapist contact. As shown in Table 1, there were no instances of significant trends (using τ) and so no therapist contact and therapist contact baselines were collapsed to form a single baseline phase for each measure. After creating these single baselines, there became significant statistical trend evident within the baseline periods of self‐criticism (worsening, τ = 0.277, *p* = .009) and mood (becoming more manic, τ = 0.251, *p* = .018). As baseline trends were either not significant or significant but showing deterioration (mood, self‐criticism), it was not necessary to perform baseline trend adjustments.

**TABLE 1:** Means, SDs, τ and overlap statistics between baseline phases

Daily measure	Pre‐assessment vs. assessment period											
	Pre‐assessment			CAT assessment			Collapsed baseline			Comparisons		
	τ	Mean	*SD*	τ	Mean	*SD*	τ	Mean	*SD*	τ^AvsB^	NAP	PEM
Self‐Criticism	−.220	6	1.3	−.010	7.38	0.62	.277*	6.93	1.1	.675*	83.74	93.10
Self‐Compassion	−.220	6.29	1.59	.239	6.17	1.23	.050	6.21	1.34	.079	39.90	3.45
Body Satisfaction	−.319	6.71	1.2	.096	6.31	0.89	−0.080	6.44	1.01	−.202	46.06	3.45
Worrying	−.220	5.57	1.55	.222	5.21	1.37	0.000	5.33	1.43	−.364	41.38	17.24
Mood	.231	3.36	1.01	−0.01	4.24	0.58	0.251*	3.95	0.844	0.517*	75.86	93.10

Note

Interpretation: Higher τ value is higher phase trend.

*Significant at *p* = <.05.

### Idiographic outcomes

**FIGURE 2:**
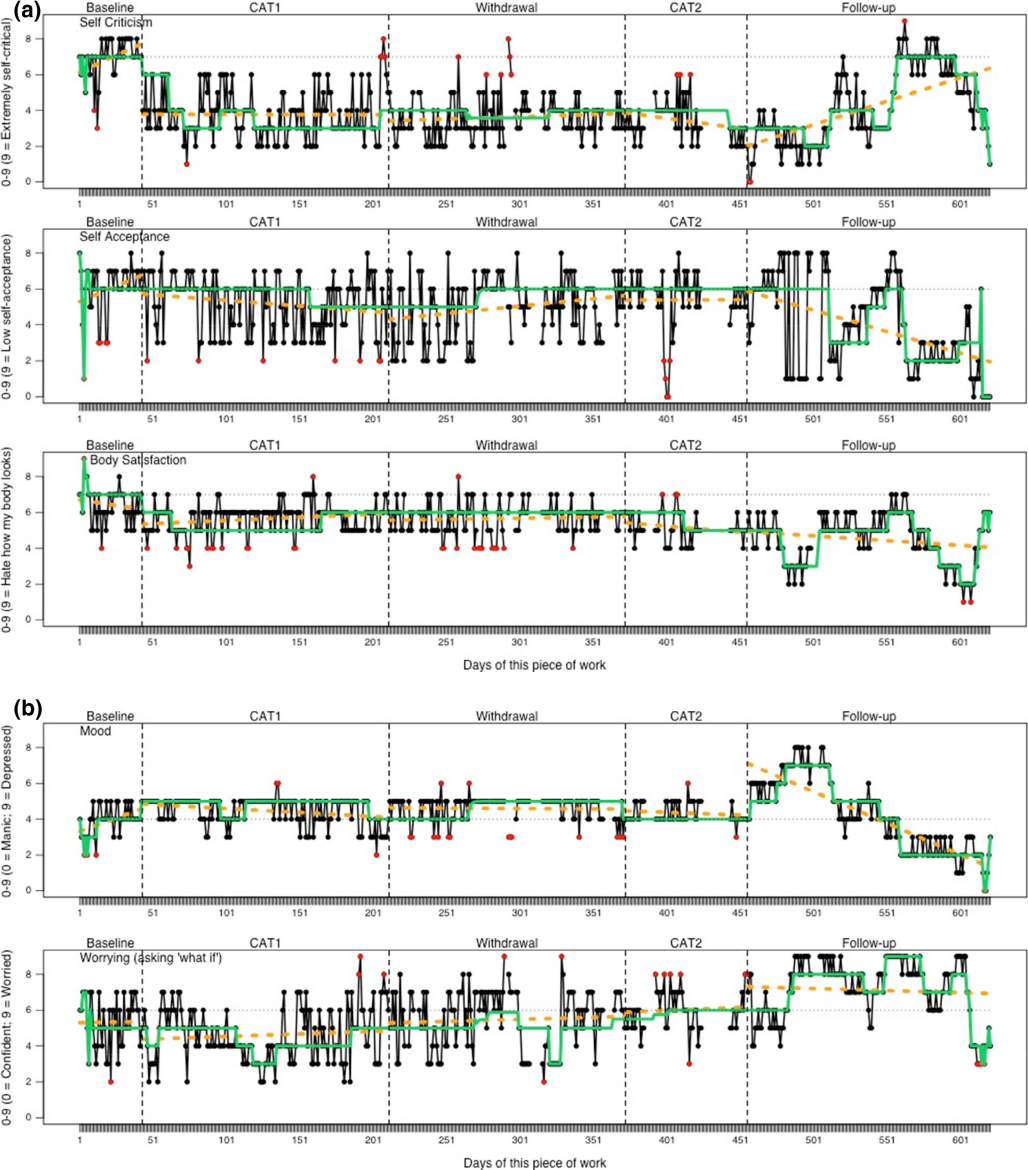
(a) Time series plots for self‐criticism, self‐compassion and body‐satisfaction idiographic measures. (b) Time series plots for mood and worry idiographic measures. *Note:* Yellow dashed lines depict phase trends. Grey horizontal dashed lines represent baseline median. Green solid line depicts the running median (lag 7). Red data points identify outlier spikes defined as points more than two SDs from the phase mean

Table 2 provides a descriptive summary (including outlier spike frequency) of the data for the idiographic measures (reported by study phase), whilst Table 3 contains the between phase comparisons (non‐overlap and τU). Figure 2a and b present the time series graphs for ideographic measures. For the measures of self‐criticism, self‐acceptance, body dissatisfaction and worry, patient improvement would be signified by a decreasing score (decrease intended measures). In contrast, for the mood measure improvement would be shown by increasing stability of score, and within the mid‐range of the Likert scale (i.e. 5 out of 9).

**TABLE 2:** Descriptive and variability statistics for each phase

Daily measure	Range, SIQR, median, *SD* and spike frequencies by phase					
	Pre‐Assessment	Assessment	CAT 1	Withdrawal	CAT 2	Follow‐up
Self‐Criticism						
Range (SIQR)	3–7 (0.88)	6–8 (0.5)	1–8 (0.5)	2–8 (0.5)	2–6 (0.5)	0–9 (1.5)
Median (*SD*)	6.5 (1.30)	7 (0.62)	4 (1.33)	3 (1.12)	3 (1.05)	4 (2.06)
Spike frequency (%)	14.29%	0%	2.98%	4.35%	3.61%	1.81%
Self‐Acceptance						
Range (SIQR)	1–8 (1)	3–8 (0.5)	2–8 (1)	2–8 (1.25)	0–8 (0.5)	0–8 (2)
Median (*SD*)	6.5 (1.94)	6 (1.23)	6 (1.52)	6 (1.66)	5 (1.64)	6 (2.42)
Spike frequency (%)	14.29%	6.90%	4.17%	0%	6.02%	0%
Body Satisfaction						
Range (SIQR)	5–9 (0.5)	4–8 (0.5)	3–8 (0.5)	4–8 (0.5)	4–7 (0.5)	1–7 (0.5)
Median (*SD*)	7 (1.20)	6 (0.89)	6 (0.82)	6 (0.86)	5 (0.81)	5 (1.35)
Spike frequency (%)	7.14%	3.45%	8.33%	8.70%	3.61%	1.20%
Worrying						
Range (SIQR)	3–7 (1.38)	2–7 (1)	2–9 (1)	2–9 (1.5)	3–8 (1)	3–9 (1)
Median (*SD*)	6 (1.56)	5 (1.37)	4 (1.46)	6 (1.62)	6 (1.08)	8 (1.67)
Spike frequency (%)	0%	3.45%	1.79%	1.86%	8.43%	3.02%
Mood						
Range (SIQR)	2–5 (0.5)	3–5 (0.5)	2–6 (0.5)	3–6 (0.5)	3–6 (0.5)	0–8 (2)
Median (*SD*)	3 (1.01)	4 (0.58)	5 (0.75)	5 (0.68)	4 (0.55)	4 (2.01)
Spike frequency (%)	21.43%	0%	1.79%	9.32%	2.41%	1.20%

Note

Spike frequency defined as percentage of phase timepoints more than 2 *SD*s from phase mean.

Abbreviations: *SD*, Standard deviation; SIQR, Semi‐interquartile range.

**TABLE 3:** Non‐overlap effect and Tau‐u statistics for ideographic measures between specific phases of SCED

Daily measure	Combined baseline vs. First CAT treatment phase			
	Baseline τ	τ^AvsB^	τ^u^	PEM
Self‐Criticism	0.277*	−0.92*	−0.827*	97.62
Self‐Compassion	0.050	−0.401*	NA	43.45
Body Satisfaction	−0.080	−0.448*	NA	88.10
Worrying	0.000	−0.287*	NA	71.43
Mood	0.251*	−0.42*	0.284*	13.10

Daily measure	Combined baseline vs. Follow‐up (B vs. FU)		First CAT treatment vs. withdrawal (B vs. W)		Combined CAT treatments vs. combined withdrawal + follow‐up (B + C) vs. (W + FU)	
	NAP	PEM	NAP	PEM	NAP	PEM
Self‐Criticism	85.92	80.72	47.97	19.26	52.65	50.83
Self‐Compassion	69.44	59.01	45.18	16.3	42.58	21.28
Body Satisfaction	87.26	96.89	50.55	14.81	40.01	8.45
Worrying	20.61	18.63	65.06	71.85	71.77	68.58
Mood	48.73	42.24	54.25	1.48	49.65	16.22

Note

*Significant at *p* = <.05.

When comparing the baseline and initial CAT treatment phase (B), significant change in level was shown across all ideographic measures. For the decrease intended measures, the direction of change was consistent with symptom improvement. Initial improvements in moving median trends were most strongly evident for self‐criticism, body satisfaction and worry (i.e. the B phase moving median consistently below baseline median) indicating an influence of the CAT treatment. Body satisfaction followed by self‐acceptance showed the most frequent intermittent data spikes away from the overall trend. Non‐overlap effect size was greatest for self‐criticism (highly effective, NAP = 94.77%), followed by self‐acceptance and body dissatisfaction (moderately effective, NAP = 70.03% and 72.40%), whilst reduced worrying was only somewhat effective (NAP = 64.37%). Significant level change was also shown for mood (increase), generating a mean average (*M* = 4.24) which was closer to the optimal mid‐point score for mood (i.e. 5 out of 9). Greater stability in mood is visually evident during the CAT treatment phase (see Figure 2b).

The impact of the withdrawal phase was considered by comparing the first CAT treatment phase with the withdrawal phase. Visually, scores within the withdrawal periods were highly comparable to adjacent CAT treatment phases (illustrated by similar phase trends and moving median lines). The exception was for worry which appeared to show minor elevation (i.e. deterioration) and increased variability during the withdrawal period. These visual observations were supported by low rates of non‐overlap (i.e. highly overlapping). A ‘somewhat’ effective change was shown for worrying (NAP = 65.06, PEM = 71.85) during the withdrawal period, suggestive of a mild deterioration. The other non‐overlap statistics comparing the first CAT treatment and withdrawal were all below 60%.

To further assess maintenance of treatment gains, the initial CAT treatment phase was compared against the follow‐up phase. First, for self‐criticism, there was considerable variability with scores ranging across the full scale. A steep worsening was shown towards the mid‐point of the follow‐up period, followed by a gradual return to the region of symptomatic improvement reached within treatment phases. For self‐acceptance, there was also variability evident, but the pattern was reversed; that is, scores increased in level (i.e., worsened) at the start of the follow‐up period, but then showed improvement approximately 50 days into the follow‐up period. However, the moving median during follow‐up remained equivalent (or below) the trend level observed in the initial CAT treatment phase (B), indicating that the short cycles of deterioration were not sustained. Body satisfaction scores showed less variability during follow‐up, initially maintaining a stable rate, with two periods of marked visible improvement occurring in the second and final quintiles of the follow‐up period. For worry, which had shown a cyclical nature throughout measurement, there was notable increase (i.e. worsening) shown during the follow‐up as indicated by both consistent elevations of level and trend lines above the initial CAT treatment phase (B) moving median and baseline median lines. This was sustained until the latter weeks of the follow‐up, at which point sudden improvement occurred. Finally, for mood, which showed sustained stability across treatment and withdrawal phase, there was an increase in variability across the follow‐up phase. There was an initial marked increase in level (indicating depressed mood) within the follow‐up, then followed by a gradual reduction. By the end of the follow‐up period, the idiographic mood measure indicated a relapse into mania. To summarize, some level of deterioration was evident during the follow‐up period for most ideographic measures; the exception was body satisfaction which showed evidence of further improvement during the follow‐up period.

### Nomothetic outcomes

**FIGURE 3:**
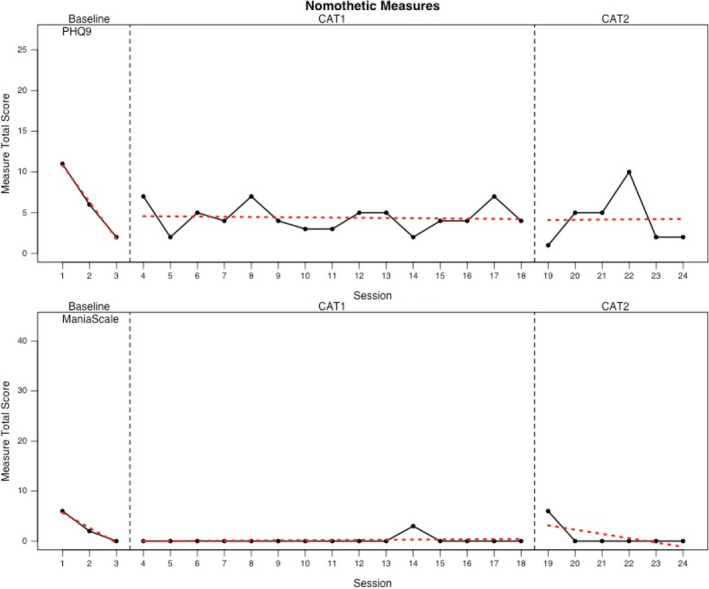
Time series plots for nomothetic measures

Scores for sessional depression (PHQ‐9) and manic mood (Mania Rating Scale) outcomes are displayed in Figure 3. Both measures improved during baseline. The vast majority of Mania Rating Scale scores 0 indexing absence of manic mood during treatment. For depression, the PHQ‐9 score at assessment indicated caseness and this had shifted to non‐caseness by the end of the baseline. During the treatment phases, this non‐case PHQ‐9 score was maintained. There was one occasion during treatment when PHQ‐9 was in the clinical range.

### Patient participant account

CAT therapy was suggested to me by my GP, one of few professionals I had encountered whom I trusted. When I began the therapy, I was traumatized by the academic and mental health experiences I’d had during my first attempt at a degree, which had resulted in dropping out. Unable to bear this, I had immediately taken a place on a different course, at a different University. This was part of a consistent behavioural theme of striving ever further in an attempt reach ‘good enough’. I had been the victim of bullying and abusive childhood and teenage friendships and had responded by avoiding developing close female friends in early adulthood. At the age of 15, I had shut myself off from sharing painful emotions with close family and friends, replacing feeling emotions with seeking academic rewards. Yet, I was unable to shut out the pain. I had spent years split in two: one part the popular, high‐achieving, intelligent graduate—physically fit and a strong climber; the other a desperately lonely young women barely able to stay afloat between long periods of traumatic mental illness. My fear of reaching out to medical professionals, close family or close friends led to an incongruence between the two halves. I had become trapped, striving for success to escape a problem I couldn't define. I felt that it had been impossible to get ‘better’ because I was a ‘walking contradiction’ of self‐confidence and self‐loathing, that I was unable to reconcile. CAT therapy, it seemed to me, was the last chance.

The narrative reformulation phase was the first time I had felt able to be truly honest with anyone about the bullying, abusive relationship I had formed with myself. Slowly, this honesty and a positive and trusting therapeutic relationships allowed me to commit, emotionally, to the diagrammatic reformulation that the therapist and I had produced together. Over the treatment period, I was able to reach out to female friends and family and develop strong reciprocal support relationships. During the treatment withdrawal phase, I learned that I was able to survive and even thrive in day‐to‐day life without constant, immediate validation from my partner. My overall mood and self‐esteem seemed better than historically, and I felt confident about the future of my mental health, in spite of the uncertainty I was facing towards the end of my PhD.

The follow‐up period took place around the time that I was graduating from my PhD, travelling to conferences, and getting married. These all represented potential triggers for mania, but techniques learned during the therapy helped to keep my feet on the ground. Checking in with myself and taking a more relaxed approach (to work and climbing in particular) were helpful—as was maintaining sleep. Towards the end of the follow‐up period, I also started a job as a researcher in industry. High external expectations combined with a controlling and critical supervisory team eventually triggered a manic state. I had gained, through viewing the completion of my PhD through a new, CAT‐tinted lens, a greater sense of personal achievement and an increased ability to ‘pace myself’ in my personal life. However, I lacked the tools to apply the CAT in a high‐pressure situation that was outside my control. Applying the same tools in a new scenario, without therapist support, proved too challenging. For a time, I struggled with the historic self‐critical thoughts, although in time I was able to recognize this, and let myself ‘off the hook’.

I came to conclude that there are situations that it is easier for me to apply the techniques formulated during CAT. Critical factors include whether the significant trigger or change is within or without my control, and whether the experience is positive or negative. With time, I felt, the tools can be well adapted to new situations without the need for further therapeutic support; the crux is that such time is not always afforded in real life.

## DISCUSSION

The purpose of this study was to evaluate the effectiveness of CAT for a patient with bipolar affective disorder in an A^1^/B/A^2^/C with follow‐up design and to co‐produce an evaluation of clinical effectiveness. No previous co‐productions of this type have been achieved. The length of the time series achieved in the study is a distinct positive feature and the methodological innovations of the split baseline and the co‐production are notable study features. The baselines were generally stable, and there was little evidence that contact with the therapist during assessment/baseline altered the idiographic outcomes. In a disorder like bipolar affective disorder where mood variability is actually part of the diagnosis (Ryle & Kerr, 2003), it is perhaps naive to expect stability during bipolar SCED baselines. The participant made particularly good use of the treatment withdrawal phase and this shows that treatment withdrawal can be an authentic aspect of the phasing of clinical interventions (Kellett et al., 2021). The introduction of the treatment withdrawal phase was clinically serendipitous, with the study originally planned as a simple A/B with follow‐up design. This shows the flexibility of SCED in meeting the changing clinical contexts, whilst also retraining methodological rigour (Barlow et al., 2009). The principle clinical concern in the current study was the observed manic relapse during the follow‐up period. Whilst consistent efforts were made to prepare the participant for the termination of therapy, the deterioration evident in the time series data raises doubts about whether CAT adequately insulated the participant from the work stress related to bullying that subsequently a triggered a bout of mood instability.

The design of the study is open to criticism; we remind readers that the design should not be seen as a true reversal design, but rather a cumulative treatment design. This is due to the fact that whilst treatment was discontinued in the third phase (A^2^, i.e. withdrawn), it was evidently (as shown in the time series plots) not reversed. A^2^ was therefore a non‐treatment/monitoring/consolidation phase and therefore conceptually different to the baseline phase (A^1^). Because psychotherapy aims for internalization and positive change that is maintainable, it clearly cannot be as cleanly withdrawn as some behavioural interventions can be (Kellett et al., 2021; McMillan & Morley, 2010). We have therefore adopted the cumulative treatment (A^1^/B/A^2^/C) description to appropriately distinguish our methodology from the conventional treatment reversal design (A^1^/B/A^2^/ B^2^), which we would consider to be less suited to interventions that are not purely behavioural and so enabling ‘clean’ withdrawal and reintroduction. The advantage of when the intervention is a medicine in a SCED study is that a ‘washout period’ (and also placebo controls) can be factored into the phases of the study, so reducing the bleed‐over bias between phases common in psychotherapeutic interventions (Lillie et al., 2011).

In terms of the innovative co‐production design, then this involved an interesting negotiation of how the methods and results could be presented that would not normally be afforded routine SCED studies. Once the participant is involved in the manner, the research is presented then this involves discussion, agreement and compromise. For example, the participant did not want their early history to be described and this needed to be respected, but were happy for TP, TPPs and SDR to be reported. Co‐production of SCED therefore mirrored to some extent the therapeutic process and because of awareness of the reformulation of the case, and then, care was taken that all decisions and stages were negotiated, so that the participant and research team were not in an enactment that mirrored the issues in the case (e.g. the repeat of bullying dynamics). It is worth noting that despite there being two active treatment phases of the study (i.e., B and C), it was the same therapy (i.e. CAT) that was being delivered, with the same narrative and diagrammatic reformulation. It is acknowledged that whilst new material might have been discussed in the final treatment phase (i.e. specifically the development and consolidation of exits), this is in keeping with the final revision phase of CAT therapy.

Overall, the results suggest a partially effective intervention. This conclusion is based on *evidence of insufficiency* in terms insulation to, and so prevention of, the manic relapse that occurred during the follow‐up in response to workplace bullying. The partially effective outcome does need to be also seen in the context of the previous lack of any clinical responsivity to a course of person‐centred counselling. This recording of a partially effective outcome contributes to the admittedly small evidence base for CAT for bipolar, which has demonstrated the promise of this brief, integrative and relational approach in helping patient's better manage extreme mood variability (Evans et al., 2017; Kerr, 2001). What the CAT appeared to primarily provide was the context for the participant to approach, process and so contain unmanageable feelings, as their previous pattern was to supress, deny and camouflage emotions. The change of self‐self relating in terms of a more balanced, compassionate and realistic model of self‐care were the sustained effects of the treatment. The recording of a partially effective outcome is also because the participant's depression was relatively mild throughout all of the first four phases (i.e. A^1^/B/A^2^/C). No changes in the PHQ‐9 were therefore evident according to phase of treatment.

The patient's attendance record suggests the high acceptability of CAT for bipolar affective disorder and so supports previous research showing CAT’s consistently low dropout rate (Calvert & Kellett, 2014; Hallam et al., 2021). The collection of such an extensive time series suggests that the close alignment of the patient and the therapist on the design of the measures enabled and ensured that the idiographic measures were not particularly burdensome (Kellett & Beail, 1997). The A^1^/B/A^2^/C design used had greater internal validity and scientific credibility than the bi‐phasic A/B single‐case designs usually employed in routine practice (Kazdin, 1978). This is because repeated change in both treatment phases compared to non‐treatment phases better tests that treatment has affected change, reducing the possibility of risk of attributing change to other factors (passage of time, regression to the mean etc.; Rizvi & Nock, 2008). Whilst the design was able to evaluate the effectiveness of the intervention, it was not possible to tease apart the impact of common factors from model specific factors. The shape of change observed in the time series graphs could therefore also reflect the impact of non‐specific factors. Whilst the patient coped better when CAT was being delivered (i.e. B and C) than when not (i.e. A^1^ and A^2^ and follow‐up), whether change was specific to CAT or to the therapeutic relationship cannot be cleaved apart. It is worth noting also that the CCAT competency measure does include items relating to the therapist displaying good common factors (Bennett & Parry, 2004).

In terms of external validity, Murad et al. (2018) would categorize the current study having low generalizability (i.e. due to concern about extending the results of the study to other bipolar patients) but high applicability (i.e. being able to draw inferences from the study regarding the psychological care of bipolar patients). More specifically, the replicability of the method of study to other bipolar affective disorder patients is open to question, although the applicability of the CAT approach to bipolar populations continues to show promise (Evans et al., 2017). Whilst the nomothetic measures and the phases of the study could be replicated with other patients, the main feature and appeal of SCED is the highly individual focus of the idiographic measures. Therefore, what made sense to the current participant to idiographically measure on an intensive daily basis over a nearly two‐year period may have little traction with another bipolar patient. Participant retention is also jeopardized the longer the intervention under investigation (Lillie et al., 2011). One method that has been attempted to increase replicability in SCED is when idiographic measures are selected by the participant from a nomothetic measure and then re‐phased for daily measurement (see Oghene et al., 2022 for an example). Therefore, the same measures in the same design across participants can be attempted, or at least the idiographic question bank come from the same nomothetic source.

The data was collected via self‐report, which limits confidence in the reliability of the results, as it raises issues of social desirability bias (Arnold & Feldman, 1981; Nicklas et al., 2010). This is particularly pertinent in a patient participant with people pleasing tendencies. Therefore, some supplementary informant data would have been useful (Kellett & Totterdell, 2013), such as the partner reporting on reducing the need to be consistently in the caring role. It is an issue that self‐acceptance and self‐criticism show contradictory trends during the follow‐up phase (increased self‐criticism alongside greater self‐acceptance). This potentially raises the issue of the reliability of the single item idiographic measures and whether the mood variability of the participant clouded perceptions of acceptance and criticism. Items may have had different meanings to the participant when in different (i.e. manic vs. euthymic/dysthymic) mood states. The wording of the idiographic measures is also an issue (e.g. ‘happy with self’ might index a more general state than the absence of self‐criticism and ‘confident’ might index a more general state than the absence of worrying). Kellett and Beail (1997) emphasized the need for collaboratively scaling idiographic measure anchors to ensure high patient centredness. It is acknowledged that the terms used to define the extremes on these scales may have introduced some measurement error or insensitivity, whilst it is certain that as they were designed by the participant, it made sense to them. The infrequent, brief spikes in scores for some of the idiographic measures may have impacted the non‐overlap statistics; however, efforts to mitigate these effects were made though the visual identification of outlier spikes, moving median trends and use of statistical effect sizes to supplement visual analysis rather than form the main basis of conclusions about effectiveness of CAT.

The nomothetic outcomes should have been collected at the follow‐up sessions and this would have helped to further index the relapse that was occurring. Additional nomothetic outcome measures may also have been also appropriate to employ. For example, the Personality Structure Questionnaire (PSQ; Pollock et al., 2001) to assess change in state‐shifting and the Scale for Suicide Ideation (e.g. the SSI; Beck et al., 1979) to more closely capture changes to dynamic risk issues. Sampling of clinical competency of the CAT intervention would have been useful also via the CCAT (Bennett & Parry, 2004). The study methodology could have been improved through the addition of idiographic control and also generalization nomothetic measures (Krasny‐Pacini & Evans, 2018). Future SCEDs with bipolar affective disorder patients may seek to utilize cross‐over designs. These designs generate fewer ethical dilemmas, due to random allocation to two different, but nevertheless active treatments (Kenward & Jones, 2014). For example, rather than withdrawing CAT, a patient could be randomly allocated to CAT followed by CBT (or vice‐versa).

To conclude, this innovative and methodologically unique study has indexed a mixed outcome of CAT for a patient with bipolar affective disorder who was treated in routine practice. The study has used a rigorous SCED methodology, which heightens the reliability and internal validity of the study, and so confidence in the conclusions drawn that CAT was partially effective. The results of SCED are of immediate benefit to the participant and the treating therapist. If enough SCEDs are completed, the participant characteristics that differentiate those that do and do not benefit from a particular psychotherapy can be explored, which allows for stratification of future patient groups in ways that would benefit outcomes. Withdrawal designs are scientifically valid but need to be carefully clinically and ethically considered prior to implementation. The length of phases of future SCED evaluations need to be matched to the extant 8‐, 16‐ or 24‐session CAT approach, and so the methodology needs to evaluate the therapy, rather than the methodology drive the therapy. This research nevertheless supports the continued testing of CAT for bipolar affective disorder and highlights the flexibility and rigour of the SCED method and the need to generate more high‐quality evidence.

## CONFLICTS OF INTEREST

The peer production design has tried to balance any conflicts of interests through collaboration during each stage of project design, analysis and reporting.

## AUTHOR CONTRIBUTION

**Stephen Kellett:** Conceptualization; Data curation; Methodology; Project administration; Writing – original draft; Writing – review & editing. **Lisa Alhadeff:** Writing – original draft; Writing – review & editing. **Chris Gaskell:** Data curation; Formal analysis; Writing – original draft; Writing – review & editing. **Melanie Simmonds‐Buckley:** Formal analysis; Software; Writing – original draft; Writing – review & editing.

## Data Availability

The R code used to analyse the data is available from the research team. Access to the data can be sought, but will be discussed with the patient participant.
